# A Remarkable Reflex

**Published:** 1900-03

**Authors:** Charles B. Kern

**Affiliations:** Lafayette, Ind.


ARTICLE VI.

### A REMARKABLE REFLEX.

CHARLES B. KERN, M. D., LAFAYETTE, IND.

I was called at 1 a. m. to see a young man twenty-four years old, who, the message said, was dying. When I reached tho house, he was trying to lie on a couch, had a flushed face and congested eyes, perspiring profusely, unable to remain in any position for more than a minute at a time, short spasmodic attempts to breathe, and complaining solely of constricted pain over the heart, over which he kept his hand constantly. Could obtain no history of the case from him, but from family learned that he had been taken very suddenly, in the manner above described, about two hours before. He had toothache in lower left molar for about a week and had used camphor freely to put on gum, and more freely than usual just before attack came on.

Gave him aconite 3x every fifteen minutes, and within one hour he was resting quietly. Saw him the following two days, feeling quite well with exception of some toothache, but with no more chest pain. Was called again the second night and found him worse than the first time. I again gave him aconite 3x, which seemed to give a slight relief, but finally was compelled to give him an opiate, as it took two men to restrain him in bed.

I now knew that the trouble must come from his tooth, for when his tooth ached, he had no other pains, and when he had chest pain his tooth felt easy. The next day I took a dentist with me, who pulled the tooth, which was an unusual one, having exostosed roots, that had extended quite deeply into the bone. His recovery was rapid after the extraction.

I give this as an unusual case of reflex pain and with the hope that it may show that reflex pains are more frequent than we imagine.—*Medical Visitor.*

